# Effect of N-terminal region of nuclear *Drosophila melanogaster* small heat shock protein DmHsp27 on function and quaternary structure

**DOI:** 10.1371/journal.pone.0177821

**Published:** 2017-05-16

**Authors:** Mohamed Taha Moutaoufik, Geneviève Morrow, Stéphanie Finet, Robert M. Tanguay

**Affiliations:** 1 Laboratoire de génétique cellulaire et développementale, Département de biologie moléculaire, biochimie médicale et pathologie, Institut de biologie intégrative et des systèmes (IBIS) and PROTEO, Université Laval, Québec, Canada; 2 IMPMC UMR7590, CNRS, UPMC Paris 6, 4 place Jussieu, Paris, France; University of Colorado Denver School of Medicine, UNITED STATES

## Abstract

The importance of the N-terminal region (NTR) in the oligomerization and chaperone-like activity of the *Drosophila melanogaster* small nuclear heat shock protein DmHsp27 was investigated by mutagenesis using size exclusion chromatography and native gel electrophoresis. Mutation of two sites of phosphorylation in the N-terminal region, S58 and S75, did not affect the oligomerization equilibrium or the intracellular localization of DmHsp27 when transfected into mammalian cells. Deletion or mutation of specific residues within the NTR region delineated a motif (FGFG) important for the oligomeric structure and chaperone-like activity of this sHsp. While deletion of the full N-terminal region, resulted in total loss of chaperone-like activity, removal of the (FGFG) at position 29 to 32 or single mutation of F29A/Y, G30R and G32R enhanced oligomerization and chaperoning capacity under non-heat shock conditions in the insulin assay suggesting the importance of this site for chaperone activity. Unlike mammalian sHsps DmHsp27 heat activation leads to enhanced association of oligomers to form large structures of approximately 1100 kDa. A new mechanism of thermal activation for DmHsp27 is presented.

## Introduction

The small heat shock proteins (sHsps) are a ubiquitous family of ATP-independent stress proteins found in all domains of life [1–5]. They are up-regulated in response to a variety of stresses that negatively impact protein homeostasis. sHsps have a low molecular weight in the range of 12–43 kDa and are able to form large oligomeric complexes with very dynamic quaternary structure [6–10].

Small Hsps contain a tripartite architecture composed of a conserved α-crystallin domain (ACD) flanked by variable N-terminal region (NTR) and C-terminal extension (CTE) [11–13]. The NTR is highly variable in length and composition and has been partially modeled; it does not show continuous electron density suggesting a certain level of structural disorder [6–8, 14, 15]. Indeed predictions suggest that the NTR contains some sequence bias common for intrinsically disordered proteins [16]. In vertebrate and plant sHsps this region has previously been described as a determinant of chaperone activity, substrate specificity and is essential for assembly of the higher order oligomers. [17–20]. The lack of sequence conservation and structure of the NTR in sHsps gives rise to the question as to whether it has a conserved functional role [21].

Phosphorylation of human sHsps has been reported to regulate their structure and function [22–27]. These sites of phosphorylation are located in the NTR suggesting that the phosphorylation primarily affects N-terminal contacts in the oligomer [20, 24]. Non-mammalian sHsps can also be phosphorylated: phosphorylated species of *Drosophila melanogaster* DmHsp27, DmHsp26, CG14207, maize Hsp22 and yeast Hsp26 have been described [28–32]. However, we ignore the phosphorylation effect of non-mammalian sHsps on their structure and activity [11].

*Drosophila melanogaster* Hsp27 (DmHsp27) is a nuclear-localized sHsp [33–35]. In addition to its up-regulation in response to stress, DmHsp27 also shows tissue- and stage-specific expression patterns during development [36]. We previously showed that, unlike metazoan sHsps, DmHsp27 forms two populations of large oligomers (725 and 540 kDa) that are able to prevent substrate aggregation [35]. Here we investigate the importance of the NTR in DmHsp27. We show that, unlike mammalian sHsps, phosphorylation of N-terminal serines in DmHsp27 does not affect the oligomeric state nor the intracellular localization of the protein.

In addition to phosphorylation sites, the NTR contains some sequences that are conserved. Deletion of a conserved FGFG motif (position F29 to G32) results in formation of large oligomers and an increase in chaperone-like activity at non-stress (non-activating) temperature using insulin as a chaperone assay. We further determined the effects of single point mutations within the FGFG motif on oligomeric structure and chaperone-like activity. Mutation of phenylalanine 29 to an alanine or tyrosine (F29A or F29Y) and glycine 30 or 32 to arginine (G30R or G32R) affects oligomerization and display a better chaperone-like activity than the wild-type protein in non-heat shock condition using insulin. Surprisingly, heat activation of DmHsp27 leads to enhanced association to form large size oligomers of approximately 1100 kDa suggest a new mechanism of thermal activation for DmHsp27.

## Results

### Effect of Serine phosphorylation on oligomeric structure and localization of DmHsp27

The NTR of vertebrate and plant sHsps has previously been described as a determinant of chaperoning activity and substrate specificity [17, 18]. At least, in vertebrate sHsps, post-translational modifications such as phosphorylation of serine residues found in the NTR are believed to affect the association/dissociation equilibrium of sHsps oligomers and lead to chaperone function activation [23, 37–39].

**Fig 1:**
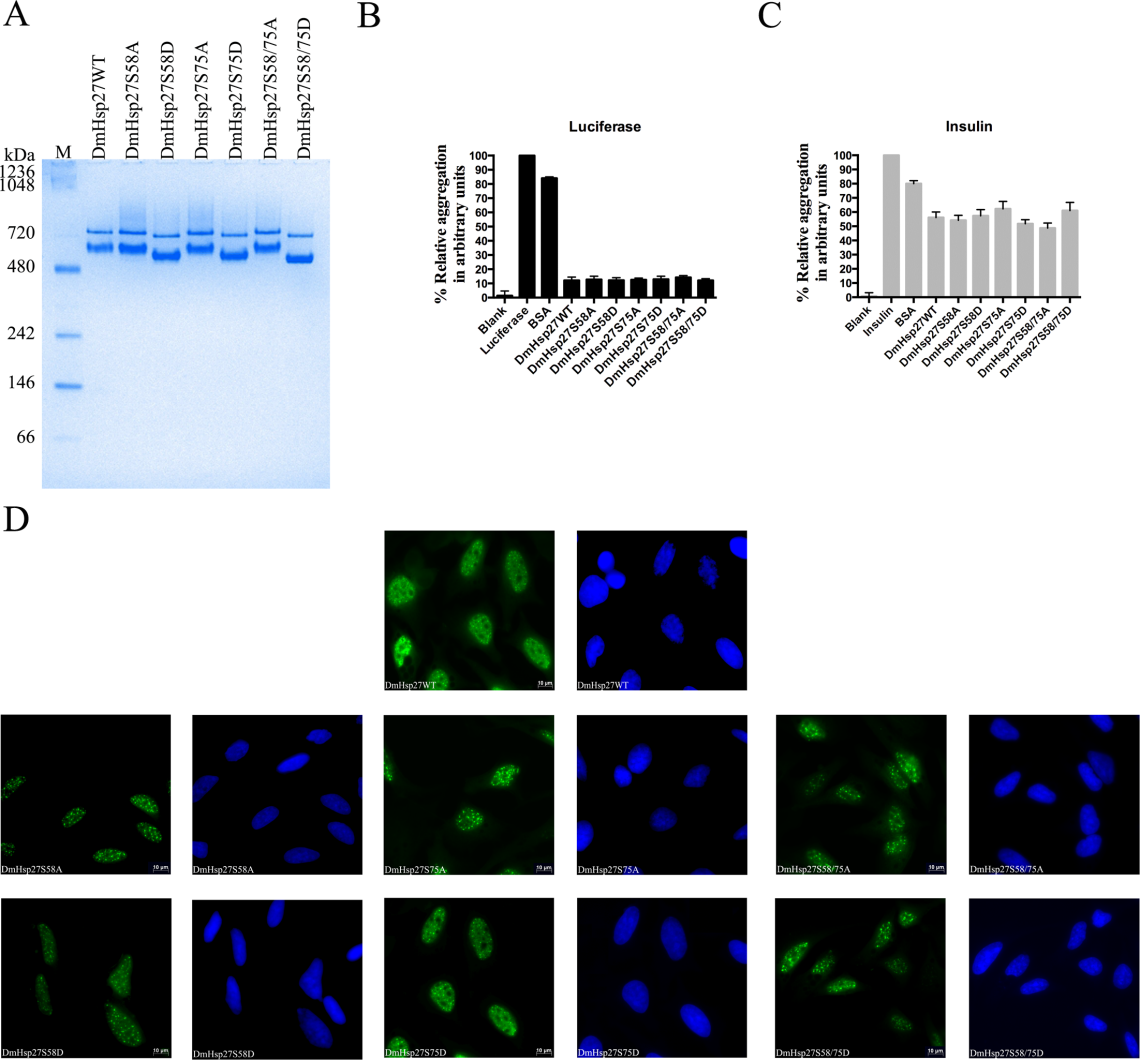
Phosphorylation effect on oligomerization and localization of DmHsp27. A- Native gradient (4–12%) polyacrylamide gel electrophoresis of recombinant DmHsp27 wild-type, phosphomimetic and nonphosphorylatable serine mutants. Positions of protein markers with molecular weights are shown on the left. B and C- Preventing aggregation of luciferase and insulin using phosphomimetic and nonphosphorylatable mutants. Data are representative of three independent experiments with error bars corresponding to the standard error of the mean. D- Intracellular localization of DmHSP27 and its phosphomimetic and nonphosphorylatable mutants in transfected Hela cells. Forty-eight hours post-transfection, HeLa cells were fixed, permeabilized, and processed for immunofluorescence using antibodies against DmHsp27 (green). Nuclei were counterstained with DAPI. Scale bar is 10 μm.

DmHsp27 is present in up to four isoforms according to the tissue and developmental stage [40]. It has been reported to be phosphorylated at NTR on at least two serines (S58 and S75) [30–32]. Whether serine phosphorylation affects the structure of DmHsp27 is an open issue. To study the effect of serine phosphorylation on the oligomeric structure and localization of DmHsp27, we constructed phosphomimetic and nonphosphorylatable mutants. To mimic phosphorylation serine (S) residues (S58 and/or S75) were substituted by aspartic acid (D) (S58D, S75D and S58/75D). To block phosphorylation, serine (S) residues (S58 and/or S75) were substituted by nonphosphorylatable alanine (A) (S58A, S75A and S58/75A). The migration profile of DmHsp27, phosphomimetic and nonphosphorylatable mutants on native gels was compared. As reported previously [35], DmHsp27 forms two populations of oligomers with apparent molecular weight of 725 and 540 kDa. Phosphomimetic and nonphosphorylatable mutants showed the same populations as wild type DmHsp27 with a light shift for phosphomimetic mutants due to the negative charge of aspartic acid (D) (Fig 1A). All mutated constructs demonstrated the equivalent chaperone-like activity to the wild type protein with different substrates (Fig 1B and 1C).

It has been reported that phosphorylation of human HspB1 and HspB5 is a mechanism for nuclear localization in unstressed cells [41]. The role of post-translational modifications on the cellular localization of DmHsp27 was therefore examined by immunofluorescence after transfection in HeLa cells. As previously reported [34, 35, 42], phosphomimetic and nonphosphorylatable mutants of DmHsp27 showed a nuclear localization and association with nuclear speckles similar to the wild type protein. (Fig 1D). These results signify that unlike vertebrate/mammalian sHsps, the two sites of phosphorylation S58 and S75 do not affect the oligomerization equilibrium nor the intracellular localization of DmHsp27.

### Further characterization of NTR in DmHsp27

**Fig 2:**
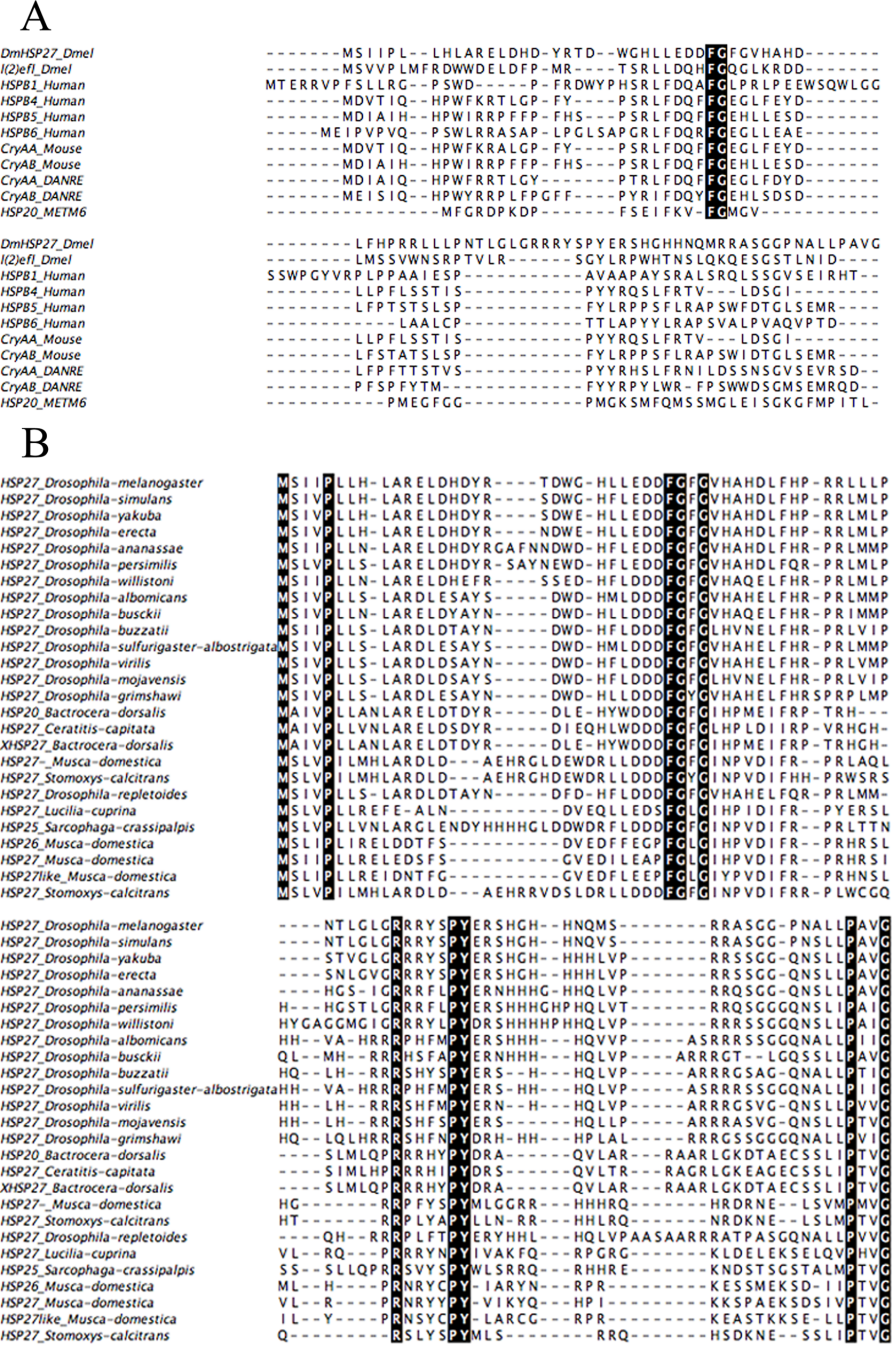
Sequence analysis of the N-terminal region of different sHsp. The alignment was made using Muscle [79]. The conserved residues are darkly highlighted in the alignment. A- Multiple sequence alignment of the NTR of DmHsp27 with l(2)efl from *Drosophila melanogaster*, human (HspB1, HspB4, HspB5 and HspB6), Mouse (CryAA and CryAB), zebrafish (CryAA_DANRE and CryAB_DANRE) and *Methanococcus maripaludis* C6 (Hsp20_METM6). B- Multiple sequence alignment of the NTR of sHsps obtained using blast similarity of NTR-DmHsp27. Sequence from *Drosophila melqnogaster*_Hsp27, *Drosophila simulans*_Hsp27, *Drosophila yakuba*_Hsp27, *Drosophila erecta*_Hsp27, *Drosophila ananassae*_Hsp27, *Drosophila persimilis*_Hsp27, *Drosophila willistoni*_Hsp27, *Dosophila albomicans*_Hsp27, *Drosophila busckii*_Hsp27, *Drosophila buzzatii*_Hsp27, *Drosophila sulfurigaster-albostrigata*_Hsp27, *Drosophila virilis*_Hsp27, *Drosophila mojavensis*_Hsp27, *Drosophila grimshawi*_Hsp27, *Drosophila repletoides_*Hsp27, *Bactrocera dorsalis*_Hsp20, Ceratitis *capitata*_Hsp27, *Bactrocera dorsalis*_Hsp27, *Musca domestica*_Hsp27, *Stomoxys calcitrans*_Hsp27, *Lucilia cuprina_*Hsp27, *Sarcophaga crassipalpis*_Hsp25.

The N-terminal region of sHsps is generally considered as being poorly conserved at the sequence level. Alignment of NTR of DmHsp27 with some human, murine, fish and *Methanococcus* sHsps shows high conservation of two residues (F29 and G30 in DmHsp27) (Fig 2A).

**Fig 3:**
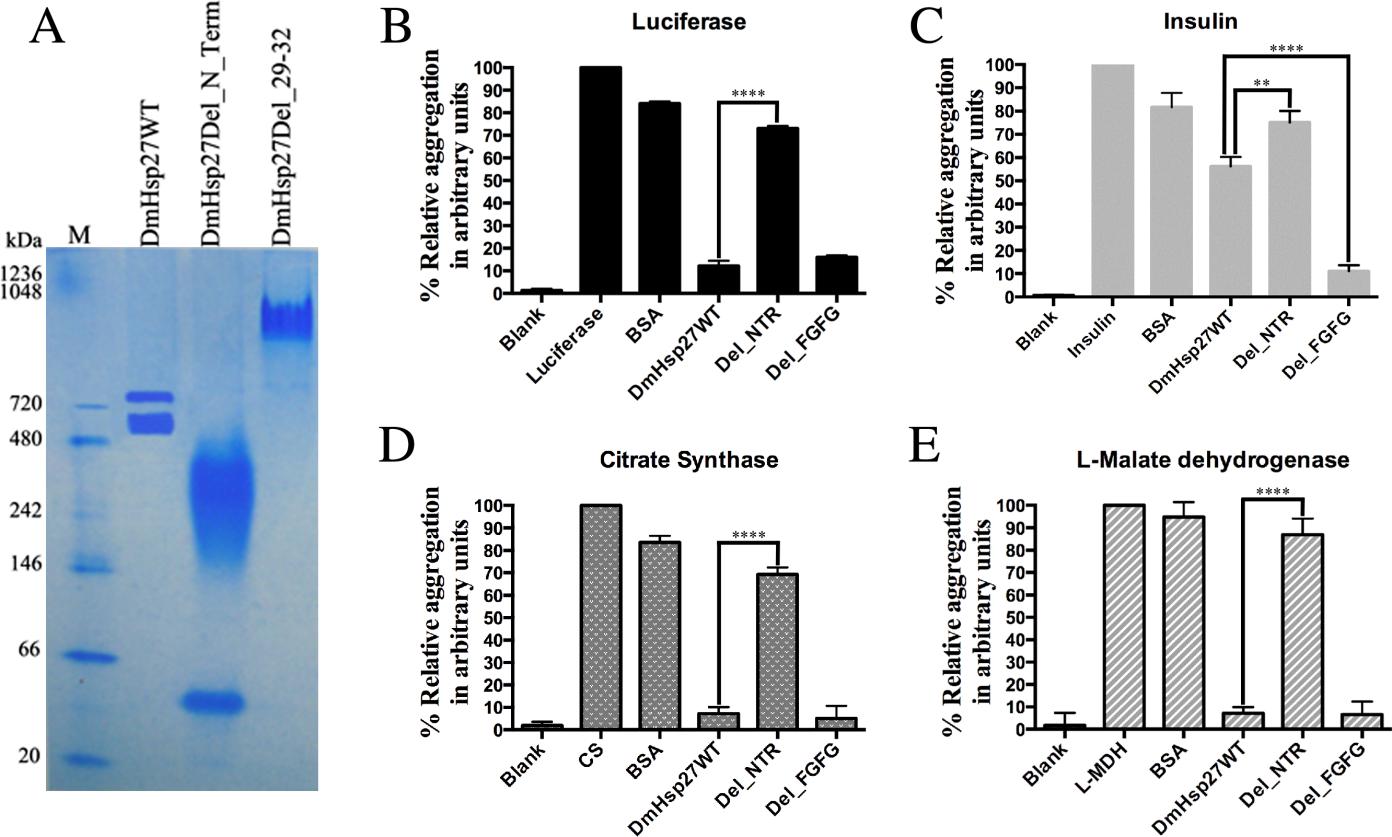
N-Terminal region is essential for oligomerization and chaperon-like activity. A- Native gradient (4–12%) polyacrylamide gel electrophoresis of recombinant DmHsp27 and mutants without N-terminal region and after deletion of FGFG motif at 20°C. B, C, D and E- Prevention of aggregation of luciferase, insulin, citrate synthase and L-malate dehydrogenase using deletion of N-terminal region or deletion of 29–32 FGFG motif. The standard error calculated from 3 sets of independent experiments. ** indicates P<0.01; **** indicates P<0.0001.

We next examined the effect of these conserved residues within the NTR on the oligomeric structure and localization of DmHsp27. A deletion of the first 86 amino acids of the N-Terminal region (Del_Nter), had a striking effect on the oligomeric equilibrium, which showed a wide band (from 480 to 146 kDa) and one extra band in the region of dimer (Fig 3A). This mutant failed to prevent heat-induced aggregation of luciferase (Luc) (Fig 3B), citrate synthase (CS) (Fig 3D), L-malate dehydrogenase (MDH), (Fig 3E) and DTT-induced aggregation of insulin (Fig 3C). Accordingly, the NTR of DmHsp27, as seen in other sHsps, is essential for oligomerization and chaperone activity at least for the substrates tested.

To further investigate residues that are important for the oligomeric structure and the chaperone-like activity of DmHsp27, we used blast similarity of NTR-DmHsp27. The obtained sequences all belong to insect sHsps. Alignment of these sequences helped to delineate a conserved sequence motif (FGXG) from phenylalanine 29 to glycine 32 in DmHsp27 (Fig 2B). Analysis of a deletion construct eliminating these four residues (del_FGFG) on a native gel, showed that the absence of this region affected the oligomeric equilibrium forming considerably large oligomers (Fig 3A). The same construct prevented heat aggregation of Luc, CS and MDH efficiently as DmHsp27 wild type (Fig 3B, 3D and 3E). Surprisingly, in non-heat condition when using insulin as a substrate, the del_FGFG construct was more efficient in prevention of DTT-induced aggregation of insulin than the WT protein (Fig 3C).

### Dissecting the FGFG (29–32) motif

**Fig 4:**
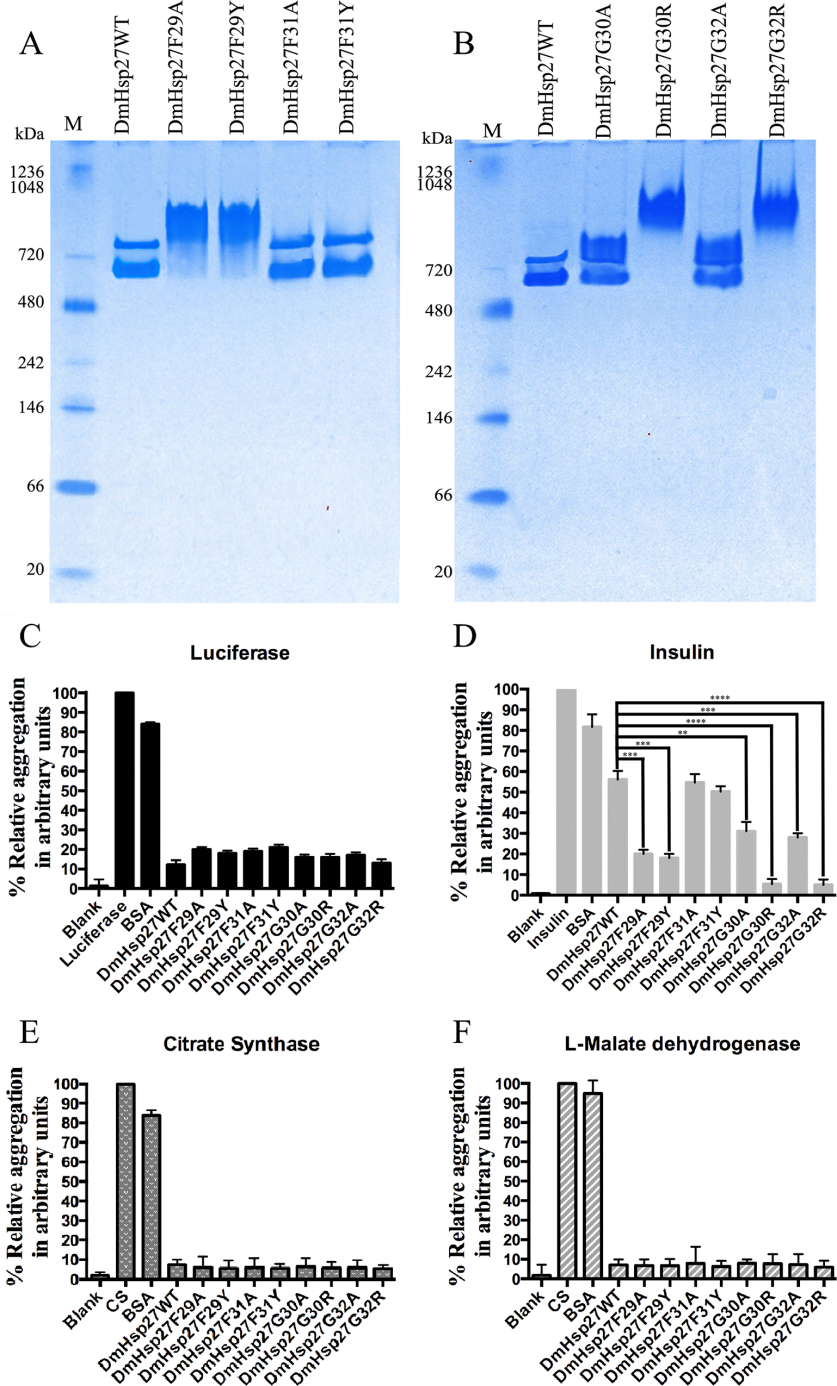
Effects of FGFG residues on oligomerization and chaperon-like activity. A and B- Native gradient (4–12%) polyacrylamide gel electrophoresis of recombinant DmHsp27 and NTR mutants (F29A; F29Y, F31A, F31Y, G30A, G30R, G32A and G32R) at 20°C. Positions of standard protein markers are shown on the left. C, D, E and F- Preventing aggregation of luciferase, insulin, citrate synthase and L-malate dehydrogenase using NTR mutants (F29A; F29Y, F31A, F31Y, G30A, G30R, G32A and G32R). Data are representatives of three independent experiments. ** indicates P<0.01; *** indicates P<0.001, **** indicates P<0.0001.

Since deletion of residues FGFG (29–32) induces formation of a large oligomer, we investigated this region more carefully using single point mutations. Specifically, we focused on F29 (present in all sequences examined) and F31 (less conserved), two residues with large side chains. Phenylalanine in both positions was mutated to an alanine, a smaller amino acid (F29A and F31A), or to an amino acid (tyrosine) that mimicked the size of the original amino acid but altered the hydrophobicity (F29Y and F31Y). The highly conserved glycines G30 and G32 were mutated to alanine (G30A and G32A) and to a positively charged larger amino acid arginine (G30R and G32R). Mutations in F31 (F31A and F31Y) (Fig 4A) or in G30 and G32 (G30A and G32A) (Fig 4B) had no effect on oligomerization as shown by the presence of two bands like wild type DmHsp27. However, F29A, F29Y, G30R and G32R showed dramatic changes in oligomeric size as seen for deletion FGFG (29–32) (Fig 4A and 4B).

**Fig 5:**
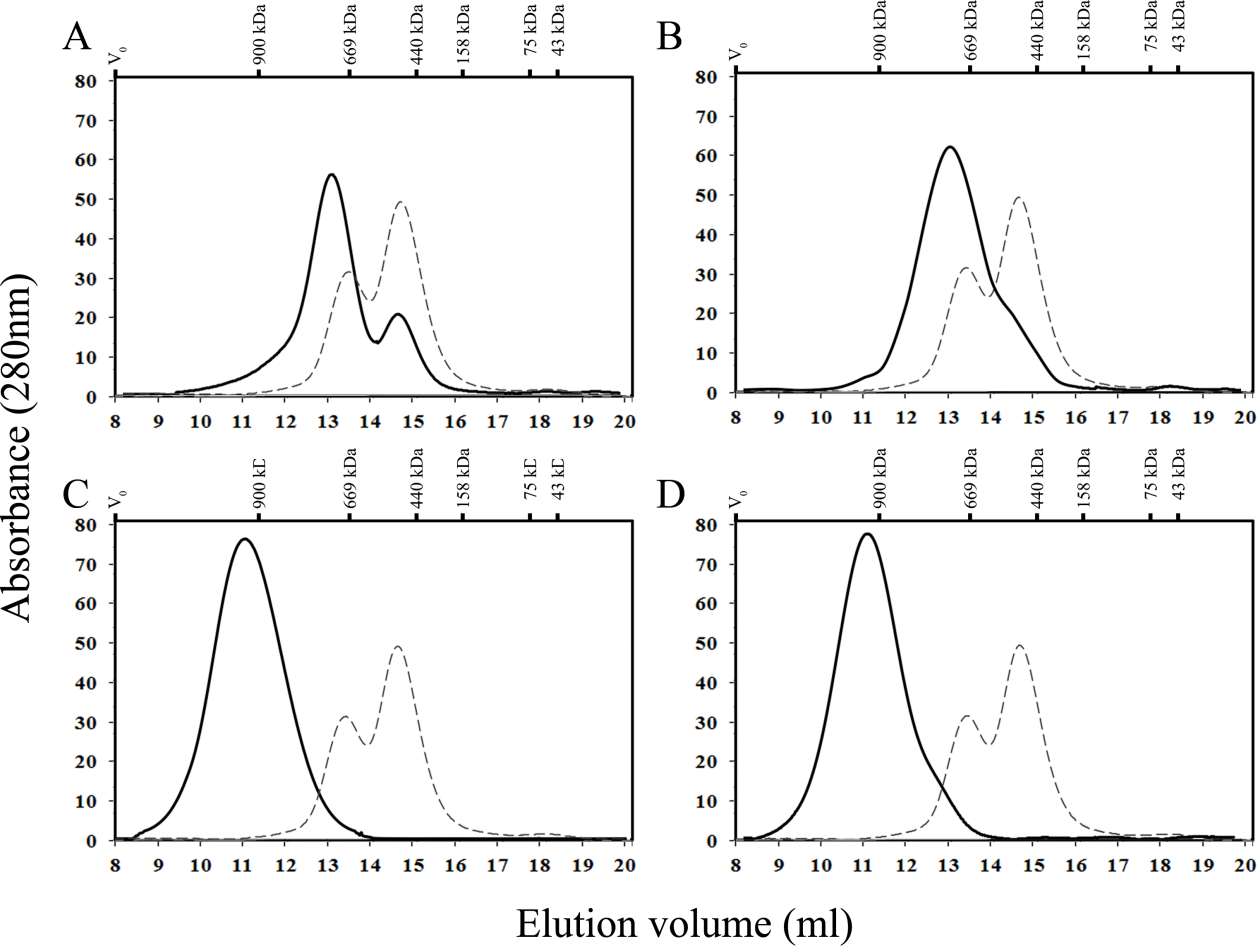
Size exclusion chromatography analysis of DmHsp27 N-terminal mutants F29A, F29Y, G30R and G32R. Size exclusion chromatography (SEC) analysis using a superose 6 10/300 GL (GE Life Sciences) column, with IGM (900 kDa), thyroglobulin (669 kDa), ferritin (440 kDa), aldolase (158 kDa), conalbumin (75 kDa) and ovalbumin (43 kDa) Blue dextran (2000 kDa) was used to determine the void volume of the column V_0_. A- Profile on column of 300 μg (black line) of DmHsp27F29A compared to 300 μg (dashed line) of DmHsp27. B- Profile on column of 300 μg (black line) of DmHsp27F29Y compared to 300 μg (dashed line) of DmHsp27. C- Profile on column of 300 μg (black line) of DmHsp27G30R compared to 300 μg (dashed line) of DmHsp27. D- Profile on column of 300 μg (black line) of DmHsp27G32R compared to 300 μg (dashed line) of DmHsp27.

The size estimation by native gel electrophoresis was confirmed using SEC for DmHsp27, F29A, F29Y, G30R and G32R mutants. As seen in Fig 5, the profile of DmHsp27F29A on Superose 6 gave two peaks (Fig 5A). The first one eluted at 13 ml and corresponded to a molecular mass of 800 kDa while the second one eluted at 14.6 ml and had a molecular mass of 540 kDa. For this mutant we noted that the peak #1 corresponding to 800 kDa was more abundant than the peak #2 of 540 kDa. For DmHsp27F29Y the profile on Superpose 6 shows one peak eluted at 13 ml (800 kDa) and a shoulder after the main peak at 14 ml (Fig 5B). While the R30G and R32G mutants show a single peak at 11ml corresponding to 1100 kDa (Fig 5C and 5D).

### Heat activation of DmHsp27

As reported previously DmHsp27WT can prevent Luc, CS, and MDH heat-induced aggregation more efficiently than insulin DTT induced aggregation [35] and Fig 4. In the same way all NTR mutants could efficiently prevent heat-induced aggregation of Luc, CS and, MDH (Fig 4C, 4E and 4F). No significant differences were shown in chaperone-like activity of DmHsp27 wild type and N-terminal mutants (F29A, F29Y, F31A, F31Y, G30A, G30R, G32A and G32R) in the heat-induced aggregation of Luc at 42°C, CS and MDH at 45°C. However, some differences in preventing aggregation using reduction of disulfide bond-induced aggregation of insulin at 20°C were observed. DmHsp27WT, F31A and F31Y mutants were less efficient. While, mutants G30R and G32R were the most efficient followed by F29A, F29Y, G30A and G32A (Fig 4D).

**Fig 6:**
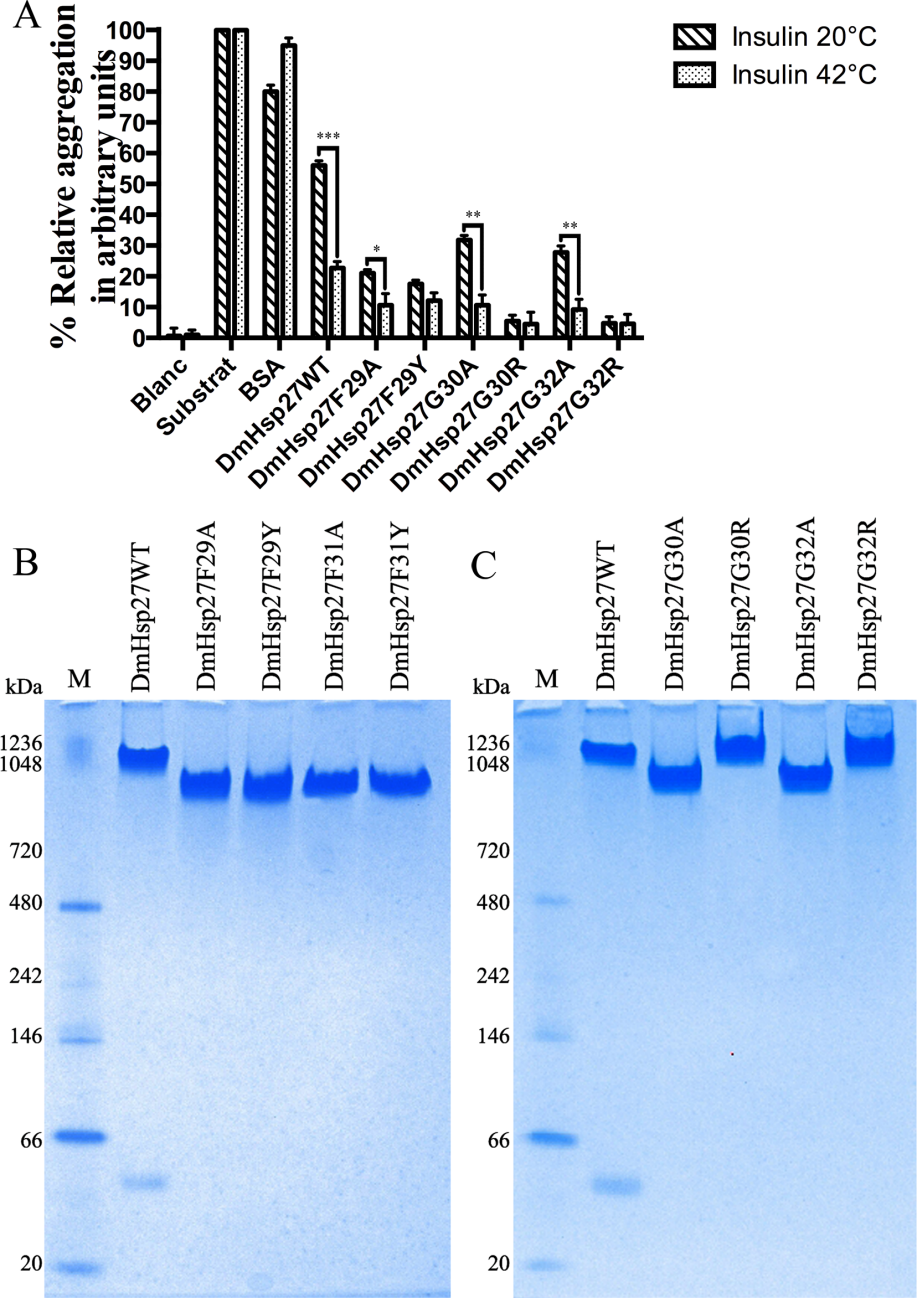
Study of heat activation of DmHsp27 and its FGFG mutants. A- Prevention of aggregation of insulin using DmHsp27WT and NTR mutants at 42°C compared to 20°C. Data are representative of three independent experiments, error bars resultant the standard error of the mean. B and C- Native gradient (4–12%) polyacrylamide gel electrophoresis of recombinant DmHsp27 and NTR mutants at 42°C. Positions of standard protein markers with known molecular weights are shown on the left. * indicates P<0.05, ** indicates P<0.01; *** indicates P<0.001.

It is possible that DmHsp27 binds more easily to Luc, CS and MDH than to the insulin substrate resulting in a stronger aggregation protection. Alternatively, heat activation of DmHsp27 could lead to a better protection. To test these hypotheses, we performed the insulin chaperone assay at 42°C. DmHsp27 and all mutants showed a high capacity to reduce insulin aggregation at 42°C compared to 20°C (Fig 6A). At 20°C DmHsp27WT prevented reduction-induced aggregation of insulin with 44% efficiency compared to 77.4% efficiency at 42°C. Mutants G30A and G32A show slight improvement at 42°C with (89.3 and 90.7% efficiency respectively) compared to 68.2 and 72.3% efficiency at 20°C (Fig 6A). No significant difference shown for F29Y, G30R and G32R mutants. We therefore compared the oligomeric structure of DmHsp27WT and NTR mutants on a native gel at 42°C. The results showed that at 42°C DmHsp27WT and NTR mutants formed oligomers of higher molecular weight than those observed at 20°C (Fig 6B and 6C). Unlike mammalian sHsps, DmHsp27 heat activation led to formation higher sized oligomers efficient to suppress aggregation of substrates.

The formation of large oligomers induced by elevated temperature are partially reversible as indicated by native gels (Supplementary S1 Fig). Interestingly, DmHsp27WT and N-terminal mutants decrease oligomeric size after recovery 30 min and 2 hours at 20°C. It should be pointed out that for DmHsp27WT, mutants F31A, F31Y, G30A and G32A two populations of oligomers appears clearly at 2 hours recovery (S1 Fig).

## Discussion

In the present work we characterized the role of a conserved motif in the NTR of DmHsp27 on its oligomeric structure and chaperone-like activity. This region is poorly conserved among sHsps and is involved in chaperoning and oligomerization.

Phosphorylation of serine residues in the NTR has been reported to regulate the chaperone activity of mammalian sHsps as recently reviewed [11, 23, 43]. A phospho-mimicking mutant of HspB1 has been found to shift the equilibrium between oligomers and dimers in favor of the smaller assemblies [39]. Similarly, studies using phosphorylation mimicking variants of HspB5 reveal an oligomer ensemble mainly consisting of 12-mers, hexamers and dimers [24]. The predominance of these smaller species indicates that the N-terminal contacts in the oligomer are influenced by phosphorylation [11]. While phosphorylation of HspB5 results in an increase in chaperone activity [24], the situation for HspB1 depends on the model substrate used [25, 37, 39, 44].

As mammalian sHsps, DmHsp27 can be phosphorylated by unknown kinase(s) on at least 2 serines in NTR (S58 and S75) [30–32]. Here we show that DmHsp27WT, and its serine 58 and 75 phosphomimetic and nonphosphorylatable mutants have the same oligomeric structure, chaperone-like activity and cellular localization. Thus unlike vertebrate/mammalian sHsps, phosphorylation of S58 and S75 in the NTR does not affect the oligomerization equilibrium nor the nuclear localization of DmHsp27.

Using molecular dynamics simulations Patel et al. [45] showed that the NTR of a sHsp dimer is flexible/dynamic and presents two major conformational forms designated “open” or “closed” suggesting that the NTR of the dimer behaves as a structural domain. Complete deletion of DmHsp27 N-terminal region leads to disruption of oligomerization and loss of chaperone function. Studies by incorporation of hydrophobic dyes, cross-linking experiments and analyses by mass spectrometry suggested that substrates bind to segments in the NTR [46–49]. Decreased of chaperone activity for DmHsp27_Del_N_Term is not linked to dissociation of oligomeric assembly but to binding role of NTR. As shown for many sHps removal of N-terminal in many sHsps leads to loss of chaperone function [18, 50–56], suggests a conserved role of N-terminal in oligomerization and chaperone-like activity.

Although, NTR has been described as poorly conserved, we delineate a conserved motif phenylalanine 29 to glycine 32 (FGFG) in orthologues of DmHsp27. This motif is equivalent to the described “the phenylalanine-rich region” in vertebrate sHsps [57]. A larger deletion that also eliminated this motif in human HspB4 and HspB5 resulted in a decrease of oligomeric size and an increase of chaperone-like activity [58]. In the case of DmHsp27 deletion of the conserved motif from phenylalanine 29 to glycine 32 (FGFG) affected the oligomeric state differently, with the formation of large oligomers, but resulted in enhancement of chaperone-like activity in non-thermal conditions using insulin. This suggests that there is at least one important residue that modulates oligomerization and chaperone-like activity in this region. We found that the less conserved residue F31 had no effect on quaternary structure nor the chaperone-like activity of DmHsp27. F29 seems important for formation of the smaller 540 kDa species seen by gel filtration [35], whereby mutation of this residue to either an alanine or tyrosine resulted in predominantly larger oligomers with molecular weights close to the 800 kDa species. G30R and G32R destabilized the balance to form a single peak corresponding to a higher oligomer with the highest chaperone-like activity in non-thermal conditions using the insulin assay.

Previous studies on the structure of Hsp16.9 from wheat [7] and Hsp16.0 from yeast [8] highlighted the important role of phenylalanines in the N-terminal region in oligomer formation and chaperone-like activity. Other studies using bovine or murine alphaB-crystallin, showed that mutation of F24, F27 or F28 decrease the oligomeric size and chaperone-like activity at elevated temperatures [59–61]. Recently, Heirbaut et al [18] showed that mutants of the conserved phenylalanine at position 33 to alanine in NTR of HspB6 was less active in insulin and yeast alcohol dehydrogenase aggregation assays and this residue was linked to the self-association properties of HspB6. F28 in bovine αB-crystallin and F33 in human HspB6, which are equivalent to F29 in DmHsp27 showed different behavior compared to F29 of DmHsp27. Interestingly mutation of glycine at position 34 to arginine in the NTR of HspB1 (equivalent to glycine at position 30 in DmHsp27) has been associated to distal hereditary motor neuropathies [62]. A study by Muranova et al [63] showed that mutant HspB1-G34R forms stable oligomers slightly larger than the corresponding oligomers of the HspB1WT and decreased chaperone-like activity.

There is significant controversy concerning the mode of activation of sHsps. sHsps reveal temperature-dependent chaperone activity in preventing aggregation of substrate proteins [64, 65]. Most chaperone activity models suggests that sHsps dissociate to small oligomeric forms, presumably dimers, which re-associate to a new oligomeric form containing the bound substrate [11, 12, 66–68]. However, some sHsps are activated differently. For example, the transition of Hsp26 from *Saccharomyces cerevisiae*, in which the transition into a state of increased substrate binding affinity and chaperone activity occurs through slight conformational changes without perturbation of the oligomeric state [69]. Another example is plant class II sHsps, that remain oligomeric but undergo structural rearrangements [70]. HspB1 exhibits heat-induced self-association, leading to increased oligomeric size, which correlates with increase in its chaperone-like activity [71]. Hsp22 (HspB8) from rat reveals heat-induced conformational changes with increased exposure of hydrophobic surfaces and chaperone-like activity [72]. *C*. *elegans* Hsp17 forms large sheet-like super-molecular assemblies (SMA) at high temperatures and only the SMA form exhibits chaperone-like activity in suppressing the aggregation of non-native substrate proteins [73]. It is clear that not all sHsps are activated by the same mechanism. In the case of DmHsp27, we observed a partial reversible heat-activation by induced structural changes that result in formation of higher oligomers of approximately 1100 kDa. DmHsp27WT is more effective as a chaperone at 42°C than 20°C using insulin. This suggests that heat enhances DmHsp27 chaperone-like activity by formation of larger oligomers.

How heat induces oligomerization of DmHsp27 is still an open question. In general, temperature-dependent conformational change in sHsps increases exposure of hydrophobic surfaces leading to increase in the chaperone-like activity by interaction with partially unfolded proteins through hydrophobic surfaces and prevents their aggregation. Two studies with alpha-crystallin from bovine lens support this proposition. First Das et al [74] showed exposure of hydrophobic surfaces at high temperature. Second, Smith et al [75] showed that hydrophobic regions around the residues 32–37 and 72–75 of αA- and 28–34 of αB-crystallin were exposed above 30°C.

In summary, this study characterized DmHsp27 mutant in the N-terminal region and we suggest a new protection mechanism played by DmHsp27 as molecular chaperone.

## Material and methods

### Cloning, expression and purification of recombinant DmHsp27

The cDNA of wild type DmHsp27 (DmHsp27WT) was cloned using GIBSON ASSEMBLY (NEB) into bacterial expression vector pETHSUK (a gift from Dr. S.Weeks, [76]) and mammalian expression vector pcDNA3.1^(+)^ at KpnI and XhoI sites by PCR as described in [35]. Mutations were introduced by using suitable oligomers (Table 1) and site-directed mutagenesis were done using Gibson assembly (NEB) and confirmed by DNA sequencing.

**Table 1:** Primers sequences used to construct DmHsp27 NTR mutants.

Primer	Primer sequences
**pETHSUK DmHsp27Fwd**	**5’-AGATTGGTGGTACCATGTCAATTATACCACTGC-3’**
**pETHSUK DmHsp27Rev**	**5’-AGCAGAAGCTTCTTACTTGCTAGTCTCCATTTTC-3’**
**pcDNA DmHsp27Fwd**	**5’-AAACTTAAGCTTGGTACATGTCAATTATACCACTGC-3’**
**pcDNA DmHsp27Rev**	**5’-CGGGCCCTCTAGACTTACTTGCTAGTCTCCATTTTC-3’**
**pETHSUK DmHsp27Del-NTRFwd**	**5’-GAACAGATTGGTGGTACAATGAAAGATGGCTTCCAG-3’**
**DmHsp27Del29-32Fwd**	**5'-GGAGGATGACGTCCATGCCCACGATCTGTTCC-3'**
**DmHsp27Del29-32Rev**	**5'-GGGCATGGACGTCATCCTCCAGCAAATGCCCCC-3'**
**DmHsp27F29AFwd**	**5’-GAGGATGACGCCGGTTTTGGCGTCCATGCCTAT-3’**
**DmHsp27F29ARev**	**5'-GACGCCAAAAGCGGCGTCATCCTCCAGC-3'**
**DmHsp27F29YFwd**	**5’-GAGGATGACTACGGTTTTGGCGTCCATGCCTAT-3’**
**DmHsp27F29YRev**	**5'-GACGCCAAAAGCGTAGTCATCCTCCAGC-3'**
**DmHsp27G30AFwd**	**5'-GAGGATGACTTCGCTTTTGGCGTCCATGCC-3'**
**DmHsp27G30ARev**	**5'- GAC GCC AAA AGC GAA GTC ATC CTC CAG C -3'**
**DmHsp27G30RFwd**	**5'- GAG GAT GAC TTC CGT TTT GGC GTC CAT GCC -3'**
**DmHsp27G30RRev**	**5'- GAC GCC AAA ACG GAA GTC ATC CTC CAG C -3'**
**DmHsp27F31AFwd**	**5'-GAGGATGACTTCGGTGCTGGCGTCCATGCC-3'**
**DmHsp27F31ARev**	**5'-GACGCCAGCACCGAAGTCATCCTCCAGC-3'**
**DmHsp27F31YFwd**	**5'-GAGGATGACTTCGGTTATGGCGTCCATGCC-3'**
**DmHsp27F31YRev**	**5'-GACGCCATAACCGAAGTCATCCTCCAGC-3'**
**DmHsp27G32A**_**Fwd**_	**5'-GACTTCGGTTTTGCCGTCCATGCCCACG-3'**
**DmHsp27G32A**_**Rev**_	**5'-GGCATGGACGGCAAAACCGAAGTCATCC-3'**
**DmHsp27G32R**_**wd**_	**5'-GACTTCGGTTTTCGCGTCCATGCCCACG-3'**
**DmHsp27G32R**_**ev**_	**5'-GGCATGGACGCGAAAACCGAAGTCATCC-3'**
**DmHsp27S58AFwd**	**5’-CGTCGTCGCTATGCGCCGTACGAGAGG-3’**
**DmHsp27S58ARev**	**5’-CCTCTCGTACGGCGCATAGCGACGCGACC-3’**
**DmHsp27S58DFwd**	**5’-CGTCGTCGCTATGACCCGTACGAGAGG-3’**
**DmHsp27S58DFwd**	**5’-CCTCTCGTACGGGTCATAGCGACGCGACC-3’**
**DmHsp27S75AFwd**	**5’-CACGTCGCGCGGCGGGAGGTCCAAACG3-’**
**DmHsp27S75ARev**	**5’-CGTTTGGACCTCCCGCCGCGCGACGTG-3’**
**DmHsp27S75DFwd**	**5’-CACGTCGCGCGGACGGAGGTCCAAACG3-’**
**DmHsp27S75DRev**	**5’-CGTTTGGACCTCCGTCCGCGCGACGTG-3’**

Fwd: forward.

Rev: reverse.

Following the detailed protocol in [35] pETHSUK DmHsp27WT and its N-terminal mutants were expressed in Escherichia coli BL21 (DE3) pLysS strain (NEB). The protein expression was induced with isopropyl-β-thiogalactoside (IPTG) (Roche).

The protein purification was accomplished by affinity chromatography using Ni-NTA agarose (Qiagen) column. His-Sumo-tag was digested with Sumo-Hydrolase followed by size exclusion chromatography (SEC) on Superose 6 10/300 column (GE Lifesciences) as described earlier [35]. All mutants gave a good yield of pure protein (25 mg/l) and could be concentrated above 15 mg/ml.

### Analysis of the quaternary structure by size exclusion chromatography

SEC was used to analyze the quaternary structure of DmHsp27. 300μg of proteins were loaded on Superose 6 10/300 column (GE Lifesciences) equilibrated with 20 mM Tris-HCl pH 8, 150 mM NaCl. SEC was achieved at room temperature and eluted at 0.5 ml/min. For estimating the molecular weight, the column was calibrated with protein markers immunoglobulin M (IGM) from bovine serum (900 kDa) (Sigma), (thyroglobulin (669 kDa), Ferritin (440 kDa), Aldolase (158 kDa), Conalbumin (75 kDa), Ovalbumin (43 kDa) and Blue Dextran 2000 to determine the void volume) (GE Lifesciences) as detailed in [35].

### Analysis of the quaternary structure by native gel electrophoresis

For native gel electrophoresis samples were kept at room temperature (20°C) or heated at 42°C using a water bath during 10 min and loaded on a gel 4–12% gradient native Tris-Glycine gels (Thermo Fisher Scientific). Gels were run at 150 V at room temperature (20°C) or at 42°C (on a water bath) using pre-cast Mini-Cell electrophoresis system (XCell SureLock, Life Technology). The protein complexes were stained with Coomassie blue immediately after electrophoresis.

### Chaperone-like activity

Luciferase, citrate synthase, L-malate dehydrogenase and insulin were used as substrates to evaluate the chaperone-like activity as as described in Morrow et al 2006 [77] and Moutaoufik et al. 2016 [35].

The heat-induced aggregation assay was performed using: luciferase (0.1 μM, Promega) at 42°C, citrate synthase (0.16 μM; Sigma) and L-malate dehydrogenase (0.65 μM; Roche) at 45°C in the absence or presence of DmHsp27 or its mutants. Altough, insulin (52 μM, Sigma) non-Heat-induced aggregation was induced by disulfide bonds reduction at 20°C. All substrats were pre-incubated alone or in the presence of DmHsp27 or its mutants and aggregation was followed by an increase in the optical density at 320 nm on a spectrophotometer with thermostated cells.

### Cell culture, transfection conditions and immunofluorescence analyses

Hela cells were maintained in MEM Alpha (Gibco) supplemented with 5% FBS. Cells were plated in advance at a confluence of 175 000 cells/well (6 well plate) containing glass coverslip for transfection. Later, cells were incubated for 4 h in OptiMEM (Gibco) containing the plasmid pcDNA3.1^(+)^-DmHsp27:Lipofectamine (Invitrogen) complex (1.5 μl Lipofectamine/1.5 μg DNA), cells were washed with culture medium and incubated for 48 h to express DmHsp27 before immunofluorescence.

Immunofluorescence was performed as described in [35, 78]. Briefly, cells were washed with PBS and fixed in methanol at − 20 C for 20 min. Cells were blocked in PBS 0.1% Tween20-X (PBST) containing 5% BSA (PBST-BSA) and were incubated one hour at room temperature with primary antibody (monoclonal anti-DmHsp27 (2C8E11) [34, 35]) diluted (1/20) in PBST-BSA. After they, were washed with PBST and were incubated 45 min with secondary antibody (goat anti-mouse Alexa 488 (Invitrogen)). Finally, cells were mounted in Vectashield mounting medium (Vector Laboratories) and examined using fluorescence microscopy (Axio Observer Z1).

## Supporting information

S1 Fig Partial reversibility after heat activation of DmHsp27 and its FGFG mutants. Native gradient (4–12%) polyacrylamide gel electrophoresis of recombinant DmHsp27 and NTR mutants heated at 42°C for 1h and cooled back at 20°C for 30 min (A) or 2h (B). Positions of standard protein markers with known molecular weights are shown on the left.
(TIF)
