# Supplementary figures and images for: Effect of N-terminal region of nuclear Drosophila melanogaster small heat shock protein DmHsp27 on function and quaternary structure
Source: PLoS One. 2017 May 16;12(5):e0177821. doi: 10.1371/journal.pone.0177821 (PMC5433770; doi:10.1371/journal.pone.0177821)

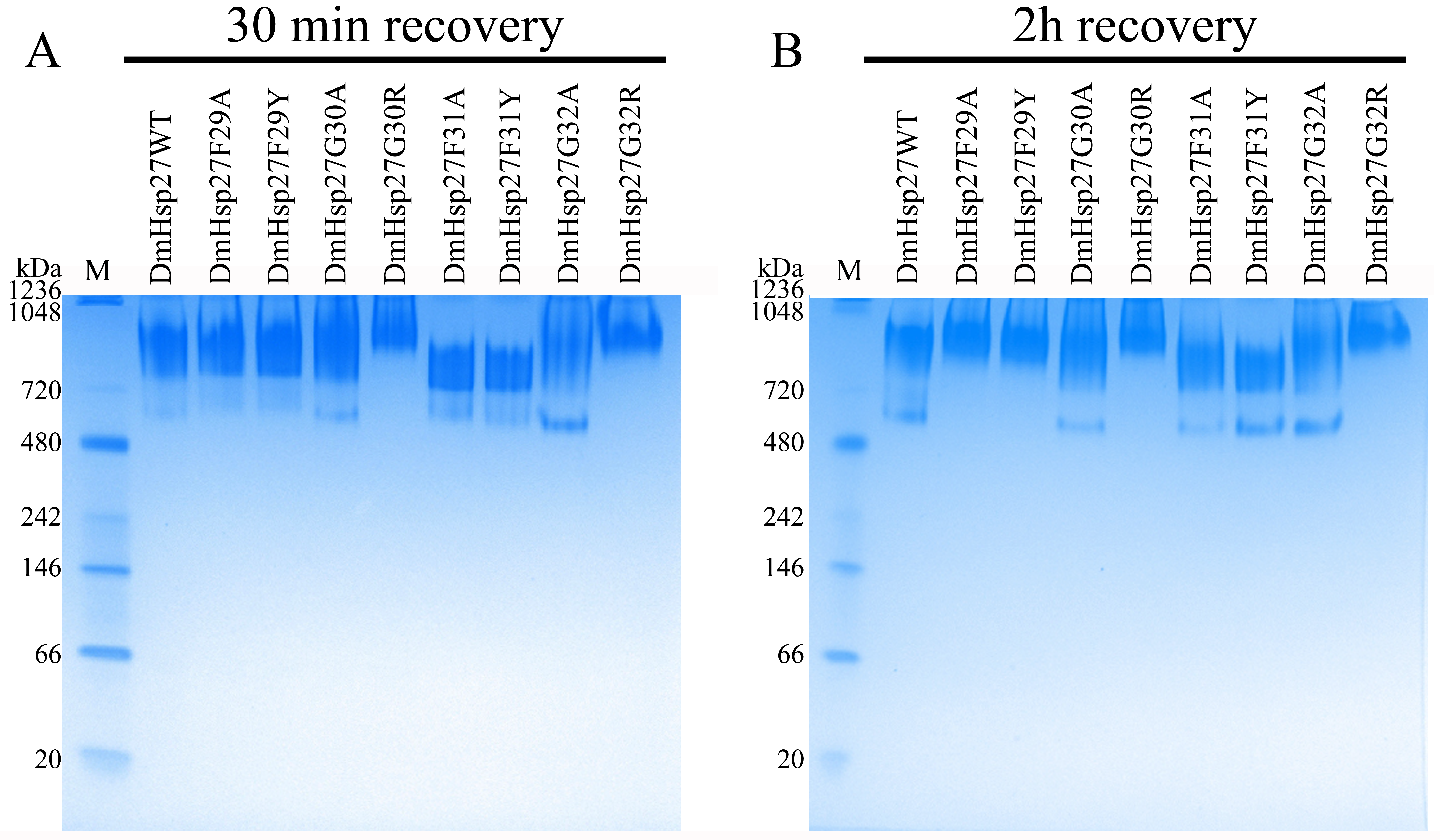

Supplement: S1 Fig — Native gradient (4–12%) polyacrylamide gel electrophoresis of recombinant DmHsp27 and NTR mutants heated at 42°C for 1h and cooled back at 20°C for 30 min (A) or 2h (B). Positions of standard protein markers with known molecular weights are shown on the left. (TIF) [file pone.0177821.s001.tif]
